# Mesenchymal stem cells’ exosomes alleviate chronic visceral pain through Nrf-2-mediated oxidative stress pathway in rats

**DOI:** 10.1097/PR9.0000000000001372

**Published:** 2025-11-19

**Authors:** Yongxiao Liu, Minjie Li, Xuzhen Lian, Xiuqing Chen, Chun Lin, Lufen Jin, Jinchao You, Yidong Lai, Libin Liu, Yixia Zhu, Jianqing Lin

**Affiliations:** a Department of Anesthesiology, Ningde Municipal Hospital, Ningde Normal University, Ningde, Fujian Province, China; b Department of Anesthesiology, First Affiliated Hospital, Fujian Medical University, Fuzhou, Fujian Province, China; c Department of Anesthesiology, National Regional Medical Center, Binhai Campus of the First Affiliated Hospital, Fujian Medical University, Fuzhou, Fujian Province, China; d Fujian Medical University, Fuzhou, Fujian, China

**Keywords:** Bone marrow mesenchymal stem cell–derived exosomes, Chronic visceral hyperalgesia, Anxiety mood, Nrf-2/HO-1

## Abstract

**Introduction:**

Irritable bowel syndrome (IBS) is a prevalent chronic functional gastrointestinal disorder characterized by visceral hypersensitivity (VH), affecting over 10% of the global population. Current treatments for IBS have notable limitations, highlighting the need for novel therapeutic strategies to address chronic visceral pain and associated comorbidities.

**Objectives:**

This study aimed to investigate whether bone marrow mesenchymal stem cell-derived exosomes (BMSC-Exos) can alleviate chronic visceral pain in IBS and to explore the underlying molecular mechanisms, with a specific focus on the Nrf-2/HO-1 oxidative stress pathway.

**Methods:**

High-quality BMSC-Exos were isolated and characterized. In vitro, their neuroprotective effects were assessed in oxidatively stressed neurons. In vivo, IBS model rats received intrathecal injections of BMSC-Exos, and their effects on visceral pain sensitivity and anxiety-like behaviors were evaluated. Spinal cord tissues were analyzed to determine modulation of the Nrf-2/HO-1 pathway.

**Results:**

In vitro studies demonstrated that BMSC-Exos effectively rescued oxidative stress-induced neuronal damage. In IBS rats, intrathecally administered BMSC-Exos were internalized by spinal cord neurons, significantly reducing visceral hypersensitivity and anxiety-like behaviors. Mechanistically, BMSC-Exos upregulated the Nrf-2/HO-1 antioxidant pathway, mitigating oxidative stress-induced neuronal damage.

**Conclusion:**

These findings demonstrate that BMSC-Exos alleviate chronic visceral pain and comorbid anxiety in IBS rats, likely through Nrf-2/HO-1-mediated oxidative stress reduction in spinal neurons. These results highlight BMSC-Exos as a promising acellular therapeutic strategy for IBS, offering potential applications for this debilitating disorder.

## Abstract 2

Bone marrow mesenchymal stem cell–derived exosomes alleviate chronic visceral pain and anxiety in rats with irritable bowel syndrome by activating Nrf-2/HO-1 and reducing oxidative stress.

## 1. Introduction

Irritable bowel syndrome (IBS), a functional gastrointestinal disorder affecting 11.2% of the global population,^27,33^ is characterized by chronic visceral hypersensitivity involving both peripheral and central sensitization mechanisms.^4,10,14,26^ These neuroplastic changes contribute to abdominal pain, bloating, and anxiety comorbidities, yet current therapies remain inadequate, underscoring the need for mechanistic insights and novel therapeutic targets.^8,21^

Bone marrow–derived mesenchymal stem cells (BMSCs) exhibit antinociceptive potential in chronic pain models by modulating oxidative stress markers, including enhancement of superoxide dismutase (SOD) activity and reduction of reactive oxygen species (ROS).^23^ However, clinical translation is hindered by risks of tumorigenicity, immune rejection, and poor cell survival.^25^ Emerging evidence suggests that BMSC-derived exosomes, nanovesicles enriched with proteins, lipids, and microRNAs, mediate therapeutic effects through paracrine signaling, offering advantages over BMSCs in stability, safety, and scalability.^5,7,13^ Although exosomes alleviate neuropathic pain and inflammation in diverse pathologies, their role in IBS-related visceral pain remains unexplored.^16,19,22^

Oxidative stress, marked by ROS overproduction, contributes to IBS pathogenesis. The Nrf-2/HO-1 pathway, a critical antioxidant defense axis, is activated under oxidative stress to upregulate SOD and catalase (CAT), thereby reducing malondialdehyde (MDA) and restoring redox balance.^11,15,24^ Although BMSCs demonstrate antioxidant properties, whether BMSC-exosomes modulate this pathway in visceral pain remains unknown.

This study investigated the effects of BMSC-Exos in chronic visceral hypersensitivity using a rat IBS model induced by colorectal distension (CRD) and glutamate-treated PC12 neuronal cultures. Exosomes isolated through ultracentrifugation were evaluated using in vivo intrathecal administration and in vitro co-culture systems. In addition, Nrf-2 knockdown was employed to elucidate the role of the Nrf-2/HO-1 axis in BMSC-Exos-mediated analgesia and anxiolysis. These findings aim to establish exosomes as a novel therapeutic strategy for IBS, bridging the gap between oxidative stress regulation and functional visceral pain management.

## 2. Materials and methods

### 2.1. Establishment and evaluation of the irritable bowel syndrome chronic visceral hypersensitivity model

The IBS chronic visceral hypersensitivity model was established in neonatal Sprague-Dawley rats (P8-14) by daily 60 mm Hg CRD for 1 minute using a colon balloon.^2^ Control rats underwent sham procedures without distension. At 6 weeks old, visceral sensitivity was assessed through electromyography (EMG) during 40 and 60 mm Hg CRD.^6^ Electromyography activity was recorded (RM6240BD) during 10-second distensions at 4-minute intervals. Percentage EMG change was calculated as [(CRD − baseline)/baseline] × 100%.

### 2.2. Histopathological processing of rat descending colon

Descending colons were fixed in 10% formalin for 24 hours, processed through graded ethanol (75%-100% with xylene steps), and paraffin-embedded. Sections (4–7 μm) were cut, mounted, dried, deparaffinized in xylene, rehydrated, and stained with hematoxylin for 5 minutes and eosin for 1 minute. Slides were dehydrated, cleared, and mounted with Permount.

### 2.3. Behavioral assessment of anxiety-like responses

In the open field test (OFT), rats were individually placed in an open-top transparent arena, and behavior was recorded for 5 minutes. Total distance traveled, time spent in the central zone, and rearing frequency were analyzed. In the elevated plus maze (EPM), rats were positioned at the junction of the open and enclosed arms. The number of entries into open arms, time spent in open arms, and head dips were recorded for 5 minutes.

### 2.4. Isolation, culture, and characterization of rat bone marrow–derived mesenchymal stem cells

Femurs and tibias were flushed with PBS to harvest bone marrow. Mononuclear cells were cultured in α-minimum essential medium at 37°C with 5% CO_2_. Nonadherent cells were removed after 24 hours, and the medium was replaced every 2 to 3 days. Immunophenotyping was performed using antibodies against CD73-PE, CD90-APC, CD105-PE-Cy7, CD34-PE, and CD45-APC. According to ISCT criteria, MSC identity was confirmed when ≥95% of cells were positive for CD73, CD90, and CD105 and <5% were positive for CD34 and CD45.

### 2.5. Isolation, characterization, and tracking of bone marrow mesenchymal stem cell–derived exosomes

Bone marrow–derived mesenchymal stem cells were precultured in serum-free medium for 12 hours, followed by incubation in 1% to 2% Exo-FBS-supplemented medium for 48 hours. Supernatants were processed by ultracentrifugation at 300*g* for 10 minutes, 2000*g* for 20 minutes, 10,000*g* for 30 minutes, and 106,000*g* for 70 minutes. Exosomes were analyzed by transmission electron microscopy (TEM) (2% uranyl acetate, 100 kV), nanoparticle tracking analysis for size distribution, and Western blot for Syntenin-1, TSG101, and Calnexin (1:1000, Abcam, Cambridge, United Kingdom). PKH26-labeled exosomes (10:5, PBS, 37°C for 30 minutes) were filtered (0.22 μm) and resuspended.

### 2.6. Intrathecal delivery and systemic pharmacological interventions

A polyethylene-10 catheter (Scientific Commodities Inc, Lake Havasu City, AZ, BB31695-PE/1) was implanted into the L4-L5 intervertebral space. Following a 7-day recovery period, rats received daily intrathecal injections of BMSC-Exos (10, 30, or 100 μg) or vehicle for 3 consecutive days. Concurrent systemic inhibition of Nrf-2 was achieved through intraperitoneal ML385 (10 mg/kg/d) for 5 days.

### 2.7. Experimental model construction and intervention strategies

PC12 cells (3 × 10^5^/well) were treated with 15 mmol/L glutamate (Sigma-Aldrich, St. Louis, MO) for 24 hours to induce oxidative stress. Control groups received PBS. For exosome intervention, PKH26-labeled BMSC-Exos were co-incubated during the final 12 hours to enable real-time uptake tracking. Nrf-2 knockdown was achieved using 4 siRNA arms: scrambled siRNA-NC and Nrf-2-targeting siRNAs (positions 1590, 666, and 1194). Silencing efficiency was validated by quantitative real-time polymerase chain reaction (qRT-PCR) at 24 to 48 hours (siRNA sequences are listed in Table 1).

**Table 1:** Small interfering sequence.

siRNA	Sense (5′-3′)	Antisense (5′-3′)
Nfe2l2-Rat-1590	GGA​GAG​GGA​AGA​AUA​AAG​UTT	ACU​UUA​UUC​UUC​CCU​CUC​CTT
Nfe2l2-Rat-666	CCG​AGU​UAC​AGU​GUC​UUA​ATT	UUA​AGA​CAC​UGU​AAC​UCG​GTT
Nfe2l2-Rat-1194	GGU​UCA​GUG​ACU​CGG​AAA​UTT	AUU​UCC​GAG​UCA​CUG​AAC​CTT
Negative control	UUC​UCC​GAA​CGU​GUC​ACG​UTT	ACG​UGA​CAC​GUU​CGG​AGA​ATT

### 2.8. Western blot analysis of protein expression

Proteins from spinal cord tissues were extracted using radioimmunoprecipitation Assay buffer, quantified using the bicinchoninic Acid assay, denatured at 95°C for 5 minutes, separated by 10% sodium dodecyl sulfate-polyacrylamide gel electrophoresis, and transferred onto polyvinylidene fluoride membranes. Membranes were blocked in 5% nonfat milk for 1 hour at room temperature, then incubated overnight at 4°C with primary antibodies: anti-Nrf2 (1:1000, Proteintech, Rosemont, IL), anti-HO-1 (1:1000, Proteintech), and anti-β-actin (1:7000, ABclonal, Woburn, MA). After washing with tris-buffered saline with Tween-20, membranes were incubated with horseradish peroxidase-conjugated secondary antibodies (1:2000, Proteintech). Signals were visualized using enhanced chemiluminescence and quantified with ImageJ, normalized to β-actin.

### 2.9. Quantitative real-time polymerase chain reaction

Total RNA was extracted from spinal cord tissues using TRIzol (Thermo Fisher, Waltham, MA). RNA purity and concentration were assessed with a Nanodrop spectrophotometer (260/280 nm). Complementary DNA (cDNA) was synthesized from 1 μg of RNA using the Evo M-MLV Reverse Transcriptase Kit (GenStar, Beijing, China). Quantitative real-time polymerase chain reaction was performed on an Applied Biosystems 7500 system with SYBR Green Master Mix under the following conditions: 95°C for 10 minutes, followed by 40 cycles of 95°C for 15 seconds and 60°C for 1 minute. Primer sequences are listed in Table 2.

**Table 2:** Primer used for quantitative real-time polymerase chain reaction detection.

Gene	Sequence (5′-3′)
Nrf-2	F: AAT​TGC​CAC​CGC​CAG​GAC​T
	R: TCA​AAC​ACT​TCT​CGA​CTT​ACC​CC
HO-1	F: CAG​CAT​GTC​CCA​GGA​TTT​GTC
	R: CCT​GAC​CCT​TCT​GAA​AGT​TCC​TC
MDA	F: TCT​GGA​CAA​ACC​TGA​GCC​CTA​A
	R: GAA​CCT​TGG​ACT​CCC​ACA​GAC​A
SOD	F: AGG​ATT​AAC​TGA​AGG​CGA​GCA​T
	R: AGC​CAC​ATT​GCC​CAG​GTC​TC
CAT	F: ATT​GCC​GTC​CGA​TTC​TCC​A
	R: AGG​GTC​CTT​CAG​GTG​AGT​TTG​T
GAPDH	F: CTG​GAG​AAA​CCT​GCC​AAG​TAT​G
	R: GGT​GGA​AGA​ATG​GGA​GTT​GCT

### 2.10. Immunofluorescence staining

Rats were perfused with 4% paraformaldehyde. Spinal cords were sectioned at 10 μm thickness, permeabilized with 0.3% Triton X-100 in PBS for 1 hour, blocked with 5% normal goat serum in PBS for 1 hour, and incubated with primary antibodies: rabbit anti-Nrf2 (1:200, Proteintech, 16396-1-AP), mouse anti-GFAP (1:300, CST, Danvers, MA, 3670), rabbit anti-Iba1 (1:10,000, Wako, Osaka, Japan, 019-19741), or mouse anti-NeuN (1:500, Millipore, Burlington, MA, MAB377). After PBS washes, sections were incubated with Alexa Fluor 488 or 594 secondary antibodies (1:500, Abcam, 2 hours), counterstained with DAPI (1 μg/mL, Sigma-Aldrich), and imaged using a Leica TCS SP5 confocal microscope.

### 2.11. Measurement of spinal cord and cellular antioxidant capacity and neuronal injury

Thoracolumbar spinal cord homogenates (10% wt/vol) were prepared from rats. Protein concentration was quantified using the Coomassie Brilliant Blue G-250 assay (A595 nm). Catalase activity (molybdate method, A007-1-1), SOD activity (WST-1, A001-3), and MDA content (TBA method, A003-1) were measured according to the protocols of the corresponding kits (Nanjing Jiancheng Bioengineering Institute). Neuron-specific enolase (NSE) levels in PC12 cells were determined using an ELISA kit (Shanghai Mbio Co, Ltd, Shanghai, China, ml206088).

### 2.12. Statistical analysis

All data are presented as mean ± standard error of the mean (mean ± SEM). Statistical analyses were performed using GraphPad Prism 8.0.3 (GraphPad Software, San Diego, CA). Comparisons between the 2 groups were conducted using a Student *t* test. For multigroup comparisons involving repeated measures (eg, paw withdrawal threshold data), 2-way analysis of variance (ANOVA) followed by a Bonferroni post hoc test was applied. One-way ANOVA with Tukey post hoc test was used for comparisons among 3 or more independent groups. Statistical significance was set at *P* < 0.05, with exact *P*-values reported for all significant differences.

## 3. Results

### 3.1. Neonatal colorectal distension induced chronic visceral hyperalgesia and anxiety-like behaviors in adult rats

**Figure 1.**
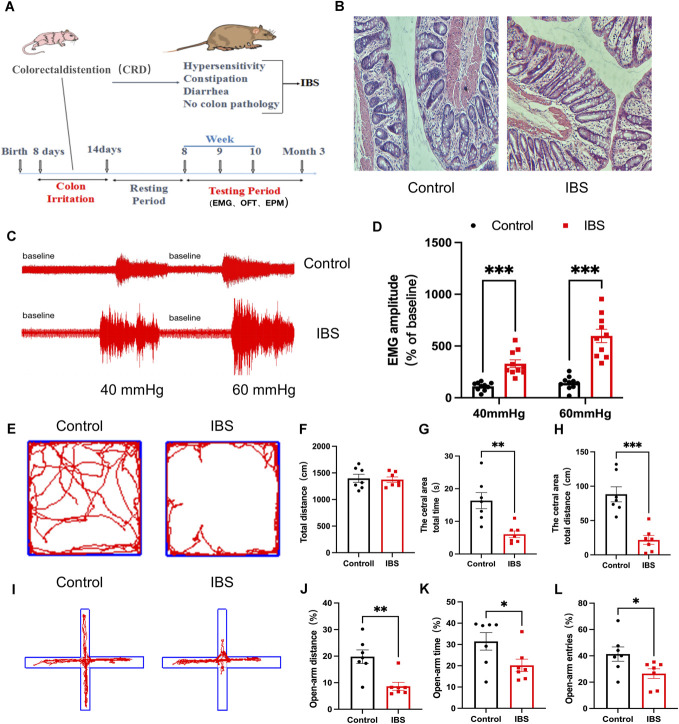
Neonatal rats exposed to CRD developed chronic visceral hyperalgesia and anxiety-like behaviors in adulthood. (A) Experimental design. (B) Hematoxylin and eosin (HE) staining of the descending colon, magnification ×100. (C and D) Representative EMG recordings in control and IBS rats under 40 and 60 mm Hg CRD stimulation, with statistical analysis of EMG amplitudes (n = 10, 2-way ANOVA, F = 52.730, ***P < 0.001). (E–H) Representative trajectories in the OFT, statistical graphs of total distance moved, time spent in the central zone, and distance traveled in the central zone in control and IBS rats (n = 7, *t* test, *t* = 3.868, *t* = 5.260, **P < 0.01, ***P < 0.001). (I–L) Representative trajectories in the EPM, statistical graphs of percentage of distance traveled in the open arms, percentage of time spent in the open arms, and percentage of open arm entries in control rats and IBS rats (n = 7, *t* test, *t* = 3.762, *t* = 2.230, *t* = 2.267, *P < 0.05, **P < 0.01). CRD, colorectal distension; EMG, electromyography; EPM, elevated plus maze; IBS, irritable bowel syndrome; OFT, open field test.

To establish an IBS rat model, male Sprague-Dawley (SD) rats were subjected to CRD beginning on postnatal day 8 for 7 consecutive days (Fig. 1A). At 8 weeks old, hematoxylin and eosin (HE) staining was performed on colonic tissues from both control and IBS rats. The results showed that, similar to the control group, the descending colon of IBS rats exhibited no marked edema, ulceration, or inflammatory cell infiltration (Fig. 1B). To evaluate whether the IBS model was successfully established, we measured the amplitude of external abdominal oblique muscle discharges during CRD. Irritable bowel syndrome rats exhibited significantly higher discharge amplitudes than control rats at 40 mm Hg and 60 mm Hg distension pressures (Figs. 1C and D), indicating visceral hypersensitivity in adulthood following neonatal CRD.

Chronic pain is frequently accompanied by negative emotions such as anxiety, which further complicate its management. To assess anxiety-like behavior, we employed 2 standard behavioral assays: the OFT and EPM. In the OFT, no significant difference in total distance traveled was observed between IBS and control rats, suggesting that neonatal CRD did not affect locomotor activity. However, IBS rats spent less time and traveled shorter distances in the central zone compared with controls (Figs. 1E–H). Consistently, in the EPM test, the percentage of time spent in and the number of entries into the open arms were significantly reduced in IBS rats (Figs. 1I–L). These findings suggest that chronic visceral hyperalgesia was associated with increased anxiety-like behaviors.

### 3.2. Imbalance of Nrf-2/HO-1 signaling in spinal cord neurons of irritable bowel syndrome rats

**Figure 2.**
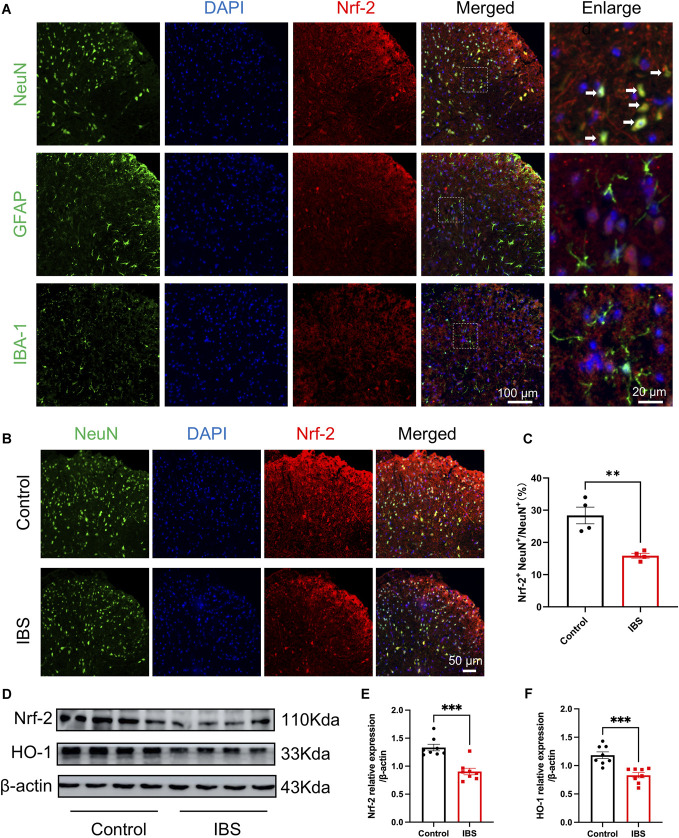
Imbalance of the Nrf-2/HO-1 signaling pathway in spinal cord neurons of IBS rats. (A) Immunofluorescence (IF) showing predominant Nrf-2 expression (red) in neurons (NeuN) in the spinal cord, with no apparent co-localization with astrocytes (GFAP) or microglia (Iba-1). DAPI was used for nuclear staining (blue). Scale bar: 100 μm (log-mag) and 20 μm (high-mag). (B and C) Representative IF images and quantitative analysis of Nrf2 in the spinal cord of control and IBS rats (n = 4, *t* test, *t* = 4.646, **P < 0.01; scale bar: 50 μm). (D–F) Representative images and statistical graphs of Nrf-2 and HO-1 protein expression in the spinal cord of control and IBS rats (n = 8, *t* test, *t* = 5.198, *t* = 4.723, ***P < 0.001). IBS, irritable bowel syndrome.

Chronic visceral pain is often accompanied by neuronal alterations, and the regulation of the antioxidative stress system plays a protective role in the development of both acute and chronic pain. To examine Nrf-2 localization in IBS model rats, double immunofluorescence staining was performed. The results showed that Nrf-2 co-localized with NeuN-positive neurons, but not with GFAP-positive astrocytes or IB1-positive microglia (Fig. 2A). Quantitative analysis demonstrated that Nrf-2 fluorescence intensity in the spinal cords of IBS rats was significantly lower than in controls (Figs. 2B and C). Western blot analysis further confirmed that protein expression levels of Nrf-2 and HO-1 were markedly reduced in IBS rats compared with controls (Figs. 2D–F). The findings suggest that disruption of the Nrf-2/HO-1-mediated antioxidative stress pathway in spinal cord neurons may be involved in IBS pathophysiology.

### 3.3. Bone marrow mesenchymal stem cell–derived exosomes reversed oxidative stress damage in neurons in vitro

**Figure 3.**
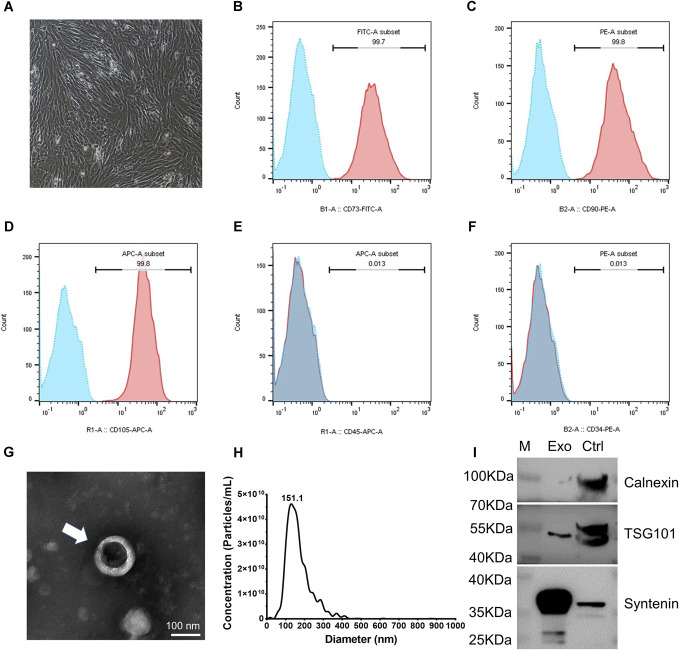
Characteristics of BMSCs and their derived exosomes. (A) Morphological characteristics of rat bone marrow mesenchymal stem cells under the microscopy, magnification ×100. (B–F) Flow cytometry histograms showing fluorescence intensity with antibodies against surface markers (red area) compared with isotype controls (blue area). CD73 (99.7%), CD90 (99.8%), and CD105 (99.8%) were positive, whereas CD34 (0.013%) and CD45 (0.013%) were negative. (G) Representative TEM image of exosomes (arrowheads). Scale bar, 100 nm. (H) Size distribution of isolated BMSC-Exos. (I) Western blot analysis of protein expression in BMSC-Exos with 293T cells as control. BMSC, bone marrow mesenchymal stem cell; TEM, transmission electron microscopy.

In this experiment, 3-week-old male SD rats served as bone marrow donors. Bone marrow–derived mesenchymal stem cells were successfully obtained by adherent culture, expanded to the third and fourth passages, and exhibited the characteristic spindle-shaped morphology (Fig. 3A). To ensure purity, flow cytometry analysis was conducted, confirming positive expression of CD73, CD90, and CD105, and negative expression of hematopoietic markers CD34 and CD45 (Figs. 3B–F). These results verified the successful isolation and identification of BMSCs.

Bone marrow mesenchymal stem cell–derived exosomes, isolated by ultracentrifugation, displayed the characteristic cup-shaped morphology under TEM (Fig. 3G). Nanoparticle tracking analysis showed that most BMSC-Exos ranged from 30 to 200 nm in diameter (Fig. 3H). Western blot confirmed the presence of classical exosome markers TSG101 and Syntenin, while Calnexin expression was absent (Fig. 3I). Together, these findings demonstrated that the extracted vesicles met the established criteria for classical BMSC-Exos.

Nrf-2 is a transcription factor that regulates the cellular antioxidant response by promoting the expression of a variety of antioxidant genes, including HO-1. Malondialdehyde, superoxide dismutase, and catalase are 3 commonly used biomarkers of oxidative stress. Malondialdehyde, a product of lipid peroxidation, is widely applied to evaluate the degree of lipid oxidation within cells. Superoxide dismutase and catalase are 2 critical intracellular antioxidant enzymes that scavenge superoxide radicals and hydrogen peroxide, respectively. Activation of the Nrf-2/HO-1 pathway enhances the expression of antioxidant enzymes such as SOD and CAT while reducing MDA production, thereby improving the cellular capacity to resist oxidative stress.

**Figure 4.**
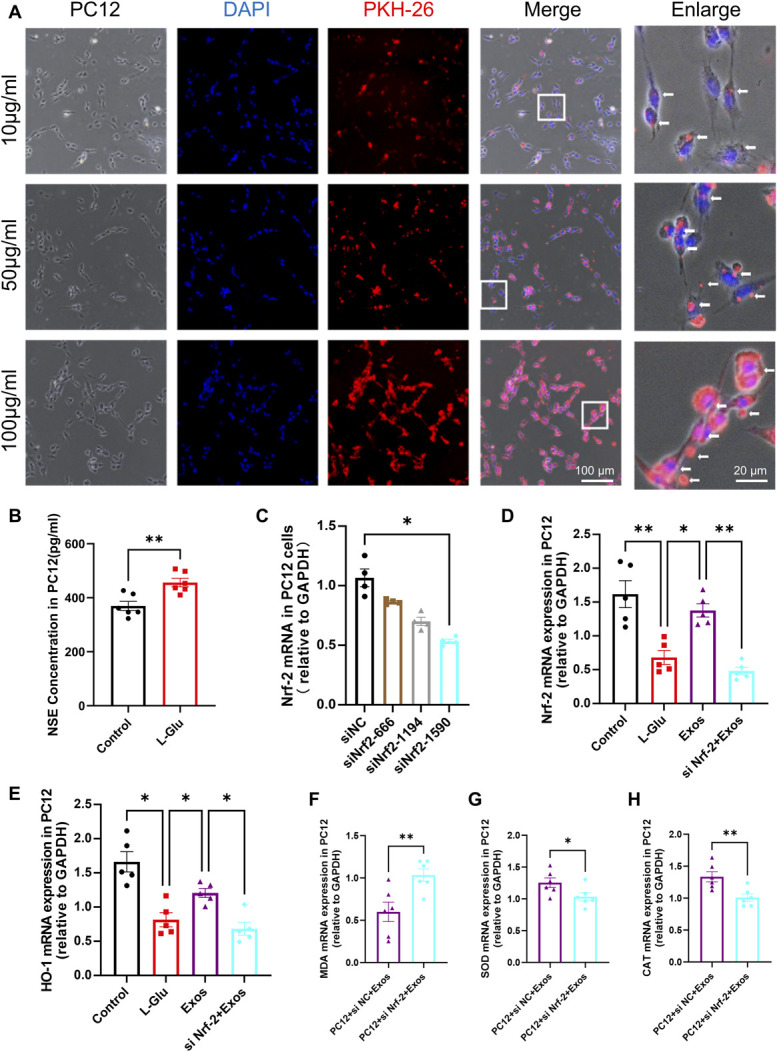
BMSC-Exos reversed oxidative stress-induced neuronal damage in vitro. (A) IF showing co-localization of different doses of PKH26-labeled BMSC-Exos (red) with PC12 cells. DAPI was used for nuclear staining (blue). Scale bar: 100 μm (log-mag) and 20 μm (high-mag). (B) Quantification of NSE expression in PC12 cells by ELISA (n = 6, *t* test, *t* = 3.758, **P < 0.01). (C) Efficiency ranking of candidate Nrf-2 siRNA at 10 nM (n = 4, 1-way ANOVA, F = 23.312, *P < 0.05). (D and E) Statistical graphs of Nrf-2 and HO-1 mRNA expression in PC12 cells across groups (n = 5, 1-way ANOVA, F = 18.886, *P < 0.05, **P < 0.01). (F–H) Statistical graphs of MDA, SOD, and CAT levels in PC12 cells in the si-NC + Exos and si-Nrf-2 + Exos groups (n = 6, *t* test, *t* = 3.118, *t* = 2.243, *t* = 3.346, *P < 0.05, **P < 0.01). BMSC, bone marrow mesenchymal stem cell; CAT, catalase; IF, immunofluorescence; MDA, malondialdehyde; NSE, neuron-specific enolase; SOD, superoxide dismutase; TEM, transmission electron microscopy.

To examine whether BMSC-Exos could rescue neuronal oxidative stress, PC12 cells were treated with 15 mmol/L glutamate for 24 hours to simulate oxidative stress injury, while control cells were treated with an equal volume of PBS. Subsequently, different concentrations of BMSC-Exos (10 µg/mL, 50 µg/mL, and 100 µg/mL) were co-cultured with PC12 cells for 12 hours, and the incorporation of BMSC-Exos into cells was observed by fluorescence microscopy. The results showed that PKH26-labeled BMSC-Exos were incorporated into PC12 cells in a concentration-dependent manner (Fig. 4A). ELISA results further demonstrated that NSE levels were increased in glutamate-treated neurons, indicating neuronal injury (Fig. 4B). These findings confirmed that BMSC-Exos were successfully internalized into oxidative stress-damaged neurons in vitro.

To clarify whether the effect of BMSC-Exos was mediated by the Nrf-2 signaling pathway, specific siRNA sequences targeting Nrf-2 transcription were designed and synthesized (Fig. 4C). Three siRNA sequences were screened in vitro, and qPCR analysis revealed that siRNA-Nrf-2-1590 was the most efficient in reducing Nrf-2 expression in PC12 cells compared with siRNA-NC, siRNA-Nrf-2-666, and siRNA-Nrf-2-1194 after transfection. Therefore, siRNA-Nrf-2-1590 was selected for subsequent experiments. PC12 cells exposed to 15 mmol/L glutamate for 24 hours were co-cultured with 50 μg/mL BMSC-Exos for 12 hours. qPCR analysis showed that Nrf-2 and HO-1 mRNA levels were reduced in oxidative stress-injured PC12 cells compared with controls, whereas this reduction was reversed by BMSC-Exos (Figs. 4D and E), suggesting that BMSC-Exos rescued neuronal oxidative stress.

Further intervention experiments demonstrated that compared with the siRNA-NC group, BMSC-Exos failed to reverse the reduction of Nrf-2 and HO-1 mRNA in damaged neurons following Nrf-2 knockdown (Figs. 4D and E); At the same time, MDA levels were significantly increased, while SOD and CAT levels were significantly decreased (Figs. 4F–H). These findings indicated that Nrf-2 knockdown abolished the ability of BMSC-Exos to rescue neurons from oxidative stress. Collectively, these results suggest that BMSC-Exos protect neurons from oxidative stress by regulating the Nrf-2/HO-1 signaling pathway in vitro.

### 3.4. Bone marrow mesenchymal stem cell–derived exosomes administration improved visceral hypersensitivity and anxiety-like behaviors in an irritable bowel syndrome rat model

**Figure 5.**
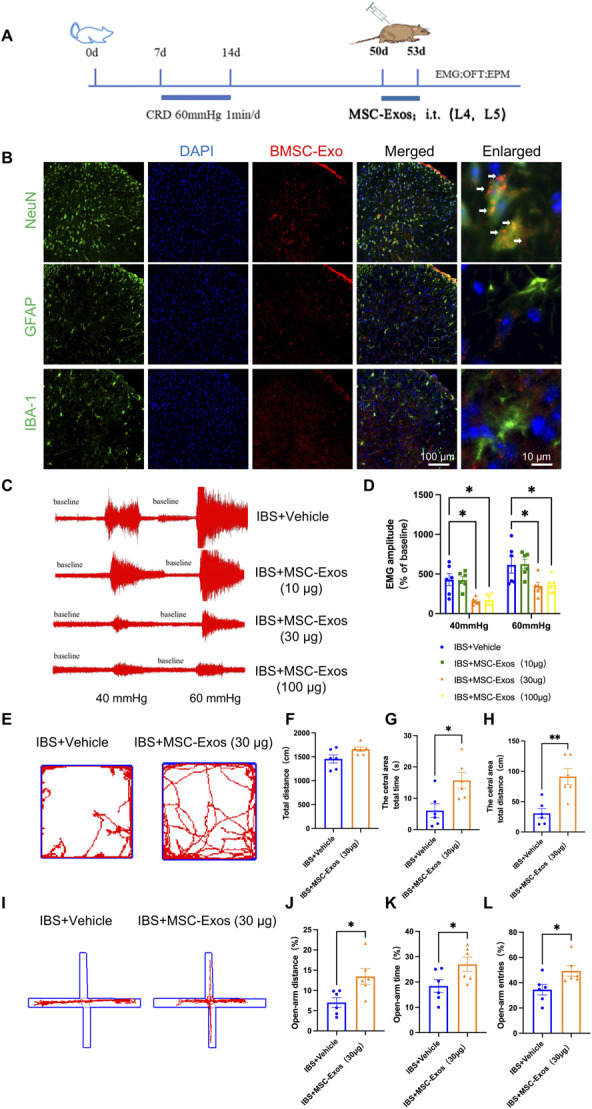
BMSC-Exos administration improved visceral hypersensitivity and anxiety-like behaviors in IBS rats. (A) Experimental design. (B) IF showing predominant localization of BMSC-Exos (red) in neurons (NeuN) of the spinal cord, with minimal co-localization in astrocytes (GFAP) or microglia (Iba-1), DAPI was used for nuclear staining (blue). Scale bar: 100 μm (log-mag) and 10 μm (high-mag). (C and D) Representative EMG recordings and statistical analysis of EMG amplitudes under 40 and 60 mm Hg CRD in rats treated with different doses (n = 6, 2-way ANOVA, *P < 0.05). (E–H) Representative OFT trajectories and statistical graphs of total distance moved, time spent in the central zone, and distance traveled in the central zone in control and exosome-injected rats (n = 6, *t* test, *t* = 2.90, *t* = 3.957, *P < 0.05, **P < 0.01). (I–L) Representative EPM trajectories and statistical graphs of percentage of distance traveled, time spent, and entries into the open arms in control rats and IBS rats treated with exosomes (n = 6, *t* test, *t* = 2.830, *t* = 2.287, *t* = 2.494, *P < 0.05). BMSC, bone marrow mesenchymal stem cell; CRD, colorectal distension; EMG, electromyography; EPM, elevated plus maze; IBS, irritable bowel syndrome; IF, immunofluorescence; OFT, open field test.

PKH26-labeled BMSC-Exos were intrathecally injected into rats at postnatal day 50 (Fig. 5A). Double immunofluorescence staining showed that BMSC-Exos were present in the dorsal horn of the spinal cord bilaterally in IBS rats, co-localizing predominantly with NeuN-positive neurons and minimal with GFAP-positive astrocytes or IBA-1-positive microglia. These findings indicated that BMSC-Exos were mainly taken up by neurons in the spinal cords of IBS rats (Fig. 5B).

To evaluate the effect of intrathecal BMSC-Exos on visceral nociceptive sensitization in IBS rats, different doses of BMSC-Exos (10 μg, 30 μg, and 100 μg) or PBS (control) were intrathecally injected on postnatal day 50. The amplitude of the external abdominal oblique muscle discharge was recorded. The results showed that 10 μg of BMSC-Exos had no significant effect, whereas both 30 μg and 100 μg significantly reduced the EMG amplitude in IBS rats (Figs. 5C and D). No significant difference was observed between the 30 μg and 100 μg doses; therefore, 30 μg was chosen for subsequent experiments. These findings demonstrated that intrathecal administration of 30 μg BMSC-Exos alleviated visceral nociceptive hypersensitivity in IBS rats.

Next, the OFT and EPM tests were used to assess anxiety-like behaviors. After 7 consecutive days of intrathecal BMSC-Exos administration, IBS + Exos rats spent significantly more time and traveled longer distances in the central zone compared with IBS + Vehicle rats (Figs. 5E–H). In the EPM test, the percentage of open arm time and entries was also significantly increased in the IBS + Exos group (Figs. 5I–L). These results indicated that intrathecal BMSC-Exos administration alleviated anxiety-like behaviors in IBS rats.

### 3.5. The antioxidative effect of bone marrow mesenchymal stem cell–derived exosomes is dependent on the Nrf-2/HO-1 pathway

**Figure 6.**
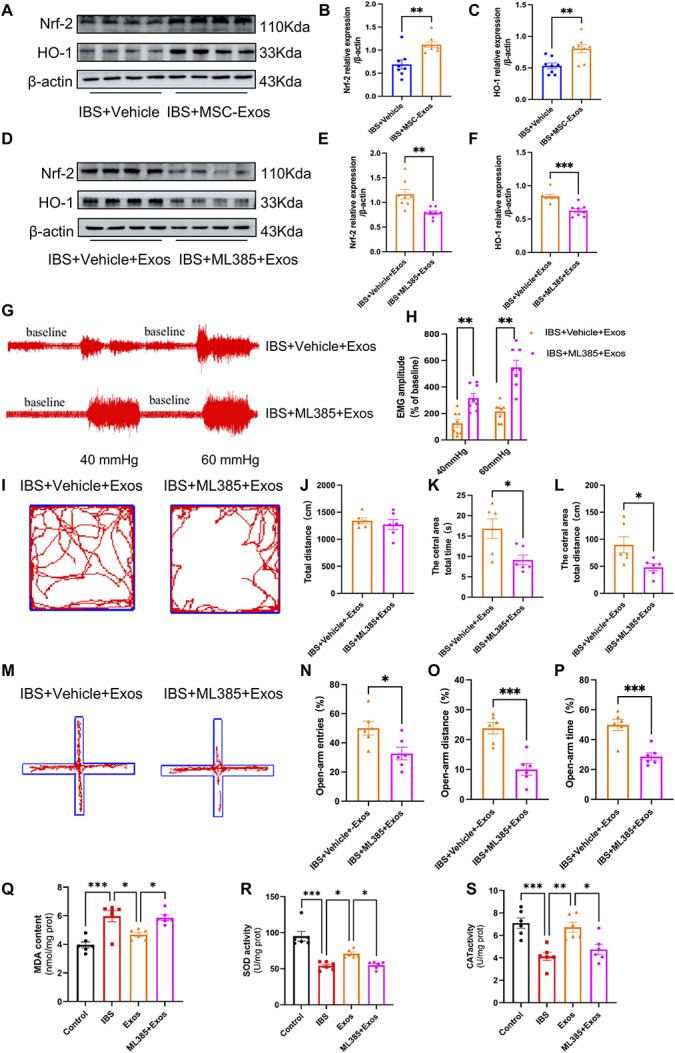
The antioxidative effect of BMSC-Exos is dependent on the Nrf-2/HO-1 pathway. (A–C) Representative images and statistical graphs of Nrf-2 and HO-1 protein expression in the spinal cord of the Model group (IBS + Vehicle) and Treatment group (IBS + BMSC-Exos) (n = 8, *t* test, *t* = 3.686, *t* = 3.867, **P < 0.01). (D–F) Representative images and statistical graphs of Nrf-2 and HO-1 protein expression in the Treatment group (IBS + Exos + Vehicle) and Antagonist group (IBS + Exos + ML385) (n = 8, *t* test, *t* = 3.655, *t* = 5.044, **P < 0.01, ***P < 0.001). (G–H) Representative EMG recordings and statistical graphs of rats in the Treatment group (IBS + Vehicle + Exos) and Antagonist group (IBS + ML385 + Exos) under 40 and 60 mm Hg CRD (n = 8, 2-way ANOVA, F = 44.790, **P < 0.01). (I–L) Representative OFT trajectories and statistical graphs of total distance moved, time spent in the central zone, and distance traveled in the central zone by rats (n = 6, *t* test, *t* = 0.682, *t* = 2.854, *t* = 2.555, *P < 0.05). (M–P) Representative EPM trajectories and statistical graphs of percentage of entries, distance traveled, and time spent in the open arms (n = 6, *t* test, *t* = 2.736, *t* = 5.085, *t* = 4.729, *P < 0.05, ***P < 0.001). (Q–S) Statistical graphs of MDA content and SOD and CAT activities in spinal cord tissues of the Blank group (Control + Vehicle), Model group (IBS + Vehicle), Treatment group (IBS + Exos), and Antagonist group (IBS + Exos + ML385) (n = 6, *t* test, *P < 0.05, **P < 0.01, ***P < 0.001). BMSC, bone marrow mesenchymal stem cell; CAT, catalase; CRD, colorectal distension; IBS, irritable bowel syndrome; MDA, malondialdehyde; OFT, open field test; SOD, superoxide dismutase.

As the Nrf-2/HO-1-mediated antioxidative pathway was suppressed in the spinal cords of IBS rats, we hypothesized that BMSC-Exos exert their therapeutic effects on chronic visceral pain through this pathway. To test this, changes in spinal Nrf-2 and HO-1 protein expression were examined after intrathecal BMSC-Exos administration. Western blot analysis showed that Nrf-2 and HO-1 protein levels were significantly increased in IBS + Exos rats compared with IBS + Vehicle rats (Figs. 6A–C).

ML385, a specific Nrf-2 inhibitor, effectively suppresses Nrf-2 activity and downstream gene expression. Rats were intraperitoneally injected with ML385 for 5 days (postnatal days 50–54) concurrently with BMSC-Exos. Compared with the BMSC-Exos group, spinal Nrf-2 and HO-1 protein expression levels were significantly reduced in the ML385 + Exos group (Figs. 6D–F). These findings suggest that the antioxidative effect of BMSC-Exos is dependent on the Nrf-2/HO-1 pathway.

To determine whether BMSC-Exos mediate visceral nociceptive sensitization and anxiety-like behaviors in IBS rats through the Nrf-2/HO-1 pathway, ML385 was preadministered intraperitoneally for 5 consecutive days from postnatal day 50 to 54, combined with intrathecal injection of 30 µg BMSC-Exos. The discharge amplitude of the external abdominal oblique muscle was measured by EMG, and anxiety-like behavior was assessed using the OFT and EPM. The results showed that the effect of BMSC-Exos in alleviating nociceptive sensitization in IBS rats was blocked by preinjection of ML385 compared with the treatment group (Figs. 6G and H). In the OFT, central time and central distance were significantly reduced in the IBS + ML385 + Exos group after ML385 administration (Figs. 6I–L). Similarly, in the EPM, preinjection of ML385 significantly reduced the percentage of open arm time and entries in the IBS + ML385 + Exos group (Figs. 6M–P).

Next, oxidative stress levels in the spinal cord were assessed. Malondialdehyde content was determined using the TBA kit, while the activities of SOD and CAT were measured using the WST-1 kit and ammonium molybdate kit, respectively. The results showed that MDA levels were significantly increased, whereas SOD and CAT activities were markedly decreased in the IBS group, indicating an imbalance of oxidative stress in the spinal cord of IBS rats. Intrathecal administration of BMSC-Exos significantly reduced MDA levels and increased SOD and CAT activities. However, this effect was abolished by preinjection of ML385 (Figs. 6Q–S). These findings suggest that BMSC-Exos enhance the resistance of spinal cord tissue to oxidative stress in IBS rats by regulating the Nrf-2/HO-1 signaling pathway.

Taken together, these results indicate that intrathecal administration of BMSC-Exos significantly alleviates chronic visceral pain sensitization and anxiety-like behaviors in IBS rats, and that these effects are blocked by preinjection of ML385. This finding suggests that BMSC-Exos mitigate chronic visceral pain and anxiety comorbidity through modulation of the Nrf-2/HO-1 signaling pathway.

## 4. Discussion

This study provides novel evidence for the therapeutic potential of BMSC-Exos in alleviating chronic visceral hyperalgesia and anxiety-like behaviors in a rat model of IBS, mediated through activation of the Nrf-2/HO-1 antioxidant pathway. These findings are consistent with the growing recognition of oxidative stress and central sensitization as critical contributors to IBS pathophysiology, while supporting BMSC-Exos as a promising cell-free therapeutic strategy.

Persistent visceral pain in IBS is increasingly attributed to central sensitization, a state of hyperexcitability in spinal and supraspinal pain-processing circuits. Our neonatal CRD model reproduced this phenomenon, with adult rats displaying exaggerated visceromotor responses and anxiety-like behaviors. These outcomes parallel clinical observations of altered pain thresholds and psychiatric comorbidities in IBS,^9^ reinforcing the interaction between peripheral nociceptive signaling and central neuroplasticity. Oxidative stress, characterized by excessive ROS and reduced antioxidant defenses, has emerged as a key driver of neuronal dysfunction in chronic pain.^29^ Our data extend this concept to IBS, demonstrating elevated MDA levels and reduced SOD and CAT activities in the spinal cord of IBS rats, suggesting that oxidative stress may impair gut-brain axis communication through both neuronal and nonneuronal mechanisms.

Recent studies highlight neuroimmune interactions as pivotal in maintaining central sensitization. For example, microglial activation in the spinal dorsal horn promotes the release of pro-inflammatory cytokines (eg, IL-1β, TNF-α), which enhance synaptic plasticity and nociceptive transmission.^18^ Although our study focused on neuronal Nrf-2/HO-1 signaling, future investigations should examine whether BMSC-Exos also modulate glial redox balance, given their reported anti-inflammatory effects in neuropathic pain models.^30^ Furthermore, the gut microbiota, a critical regulator of oxidative stress and visceral sensitivity, may interact with BMSC-Exos to influence outcomes.

The therapeutic effects of BMSC-Exos demonstrated here build upon their established roles in regulating inflammation, oxidative stress, and neuronal repair. Unlike parent BMSCs, exosomes circumvent risks of tumorigenicity and immune rejection, while retaining bioactive cargo such as miRNAs and enzymes capable of modulating recipient cells’ function.^1^ In vitro, BMSC-Exos rescued glutamate-induced neuronal oxidative damage, corroborating previous findings of their neuroprotective properties in neuropathic pain models.^31^ More importantly, intrathecal administration of BMSC-Exos attenuated both visceral hyperalgesia and anxiety-like behaviors in vivo, underscoring their dual ability to target sensory and affective dimensions of IBS. This dual efficacy contrasts with conventional therapies, such as antispasmodics and antidepressants, which often address symptoms in isolation.

A central finding of this study is the identification of the Nrf-2/HO-1 axis as the primary mediator of BMSC-Exos' therapeutic effects. Activation of Nrf-2 by BMSC-Exos led to upregulation of HO-1, SOD, and CAT, accompanied by reductions in MDA and behavioral hypersensitivity, consistent with the pathway's role in counteracting oxidative damage.^12^ Nevertheless, the observed reduction in total Nrf-2 levels in our study suggests alterations in its cellular pool but does not directly demonstrate decreased transcriptional activity. Moreover, although our findings suggest that the Nrf-2/HO-1 pathway in recipient cells is essential for the protective effects of BMSC-Exos, whether Nrf-2 knockdown influences exosome uptake efficiency remains an important question for future research.

Despite these promising findings, several limitations should be acknowledged. First, the neonatal CRD model replicates visceral hypersensitivity but does not fully capture the heterogeneity of human IBS, which also involves dysbiosis, immune activation, and postinfectious mechanisms.^3,17,31^ Integration of multiomics approaches, such as metagenomics and metabolomics, may elucidate how BMSC-Exos interact with host-microbiota networks. Second, while intrathecal administration was effective for mechanistic studies, it has limited clinical applicability. Alternative delivery routes, such as intravenous or oral administration, which have recently been optimized for exosome-based therapies using nanoparticle encapsulation^32^ should be evaluated for bioavailability and gut-brain axis targeting. Third, the contribution of specific exosomal cargo, such as miR-23a-3p or miR-27a, to Nrf-2 activation remains undefined. Proteomic or miRNA sequencing of BMSC-Exos could identify candidate mediators, enabling the design of engineered exosomes with enhanced therapeutic specificity. For instance, exosomes enriched with Nrf-2-inducing miRNAs, such as miR-34a, have shown efficacy in neurodegenerative disease models.^20^ Finally, electrophysiological studies are warranted to determine how BMSC-Exos influence synaptic plasticity in spinal dorsal horn neurons, a critical determinant of central sensitization.

This study demonstrates that BMSC-Exos ameliorate chronic visceral hyperalgesia and anxiety-like behaviors in IBS rats through neuron-specific activation of the Nrf-2/HO-1 pathway. By targeting oxidative stress and central sensitization, BMSC-Exos provide a novel, cell-free therapeutic approach for IBS that addresses both nociceptive and psychiatric comorbidities. Future research should incorporate gut-brain axis components, optimize delivery systems, and apply exosome engineering to advance clinical translation. Such efforts may transform therapies not only for IBS but also for other functional pain disorders associated with redox imbalance and neuroplasticity, including fibromyalgia and migraine.

## Disclosures

The authors have no conflict of interest to declare.

## References

[1] Baglio SR Rooijers K Koppers-Lalic D Verweij FJ Pérez Lanzón M Zini N Naaijkens B Perut F Niessen HW Baldini N Pegtel DM. Human bone marrow- and adipose-mesenchymal stem cells secrete exosomes enriched in distinctive miRNA and tRNA species. Stem Cell Res Ther 2015;6:127. 26129847 10.1186/s13287-015-0116-z PMC4529699

[2] Chen Y Chen AQ Luo XQ Guo LX Tang Y Bao CJ Lin L Lin C. Hippocampal NR2B-containing NMDA receptors enhance long-term potentiation in rats with chronic visceral pain. Brain Res 2014;1570:43–53. 24824341 10.1016/j.brainres.2014.05.001

[3] Chen Y Feng S Li Y Zhang C Chao G Zhang S. Gut microbiota and intestinal immunity—a crosstalk in irritable bowel syndrome. Immunology 2024;172:1–20. 38174581 10.1111/imm.13749

[4] Davis DA Ghantous ME Farmer MA Baria AT Apkarian AV. Identifying brain nociceptive information transmission in patients with chronic somatic pain. Pain Rep 2016;1:e575. 28503674 10.1097/PR9.0000000000000575 PMC5424698

[5] Evangelista AF Vannier-Santos MA de Assis Silva GS Silva DN Juiz PJL Nonaka CKV Dos Santos RR Soares MBP Villarreal CF. Bone marrow-derived mesenchymal stem/stromal cells reverse the sensorial diabetic neuropathy via modulation of spinal neuroinflammatory cascades. J Neuroinflammation 2018;15:189. 29933760 10.1186/s12974-018-1224-3 PMC6015468

[6] Fan F Tang Y Dai H Cao Y Sun P Chen Y Chen A Lin C. Blockade of BDNF signalling attenuates chronic visceral hypersensitivity in an IBS-like rat model. Eur J Pain 2020;24:839–50. 31976585 10.1002/ejp.1534 PMC7154558

[7] Gold MS Gebhart GF. Nociceptor sensitization in pain pathogenesis. Nat Med 2010;16:1248–57. 20948530 10.1038/nm.2235 PMC5022111

[8] Guo W Wang H Zou S Gu M Watanabe M Wei F Dubner R Huang GT Ren K. Bone marrow stromal cells produce long-term pain relief in rat models of persistent pain. Stem Cells 2011;29:1294–303. 21630378 10.1002/stem.667 PMC3277433

[9] Hanna-Jairala I Drossman DA. Central neuromodulators in irritable bowel syndrome: why, how, and when. Am J Gastroenterol 2024;119:1272–84. 38595149 10.14309/ajg.0000000000002800 PMC11208063

[10] Hosseini M Yousefifard M Aziznejad H Nasirinezhad F. The effect of bone marrow-derived mesenchymal stem cell transplantation on allodynia and hyperalgesia in neuropathic animals: a systematic review with meta-analysis. Biol Blood Marrow Transplant 2015;21:1537–44. 25985918 10.1016/j.bbmt.2015.05.008

[11] Itoh K Chiba T Takahashi S Ishii T Igarashi K Katoh Y Oyake T Hayashi N Satoh K Hatayama I Yamamoto M Nabeshima Y. An Nrf2/small Maf heterodimer mediates the induction of phase II detoxifying enzyme genes through antioxidant response elements. Biochem Biophys Res Commun 1997;236:313–22. 9240432 10.1006/bbrc.1997.6943

[12] Jiang W Yu W Tan Y. Activation of GPR55 alleviates neuropathic pain and chronic inflammation. Biotechnol Appl Biochem 2025;72:196–206. 39219239 10.1002/bab.2656

[13] Lee MJ Yoon TG Kang M Kim HJ Kang KS. Effect of subcutaneous treatment with human umbilical cord blood-derived multipotent stem cells on peripheral neuropathic pain in rats. Korean J Physiol Pharmacol 2017;21:153–60. 28280408 10.4196/kjpp.2017.21.2.153 PMC5343048

[14] Liu L Hua Z Shen J Yin Y Yang J Cheng K Liu A Wang L Cheng J. Comparative efficacy of multiple variables of mesenchymal stem cell transplantation for the treatment of neuropathic pain in rats. Mil Med 2017;182:175–84. 28291470 10.7205/MILMED-D-16-00096

[15] Otterbein LE Mantell LL Choi AM. Carbon monoxide provides protection against hyperoxic lung injury. Am J Physiol Lung Cell Mol Physiol 1999;276:L688–94. 10.1152/ajplung.1999.276.4.L688 10198367

[16] Poongodi R Yang TH Huang YH Yang KD Chen HZ Chu TY Wang TY Lin HC Cheng JK. Stem cell exosome-loaded Gelfoam improves locomotor dysfunction and neuropathic pain in a rat model of spinal cord injury. Stem Cell Res Ther 2024;15:143. 38764049 10.1186/s13287-024-03758-5 PMC11103960

[17] Porcari S Ingrosso MR Maida M Eusebi LH Black C Gasbarrini A Cammarota G Ford AC Ianiro G. Prevalence of irritable bowel syndrome and functional dyspepsia after acute gastroenteritis: systematic review and meta-analysis. Gut 2024;73:1431–40. 39013599 10.1136/gutjnl-2023-331835

[18] Rangel-Gomez M Alberini CM Deneen B Drummond GT Manninen T Sur M Vicentic A. Neuron-glial interactions: implications for plasticity, behavior, and cognition. J Neurosci 2024;44:e1231242024. 39358030 10.1523/JNEUROSCI.1231-24.2024 PMC11450529

[19] Ren J Liu N Sun N Zhang K Yu L. Mesenchymal stem cells and their exosomes: promising therapeutics for chronic pain. Curr Stem Cell Res Ther 2019;14:644–53. 31512998 10.2174/1574888X14666190912162504

[20] Shah S Mansour HM Aguilar TM Lucke-Wold B. Mesenchymal stem cell-derived exosomes as a neuroregeneration treatment for Alzheimer's disease. Biomedicines 2024;12:2113. 39335626 10.3390/biomedicines12092113 PMC11428860

[21] Shi Y Wang Y Li Q Liu K Hou J Shao C Wang Y. Immunoregulatory mechanisms of mesenchymal stem and stromal cells in inflammatory diseases. Nat Rev Nephrol 2018;14:493–507. 29895977 10.1038/s41581-018-0023-5

[22] Shipman WD Fonseca R Dominguez M Bhayani S Gilligan C Diwan S Rosenblum D Ashina S Tolba R Abd-Elsayed A Kaye AD Hasoon J Schatman ME Deer T Yong J Robinson CL. An update on emerging regenerative medicine applications: the use of extracellular vesicles and exosomes for the management of chronic pain. Curr Pain Headache Rep 2024;28:1289–97. 39495409 10.1007/s11916-024-01309-4

[23] Shiue SJ Rau RH Shiue HS Hung YW Li ZX Yang KD Cheng JK. Mesenchymal stem cell exosomes as a cell-free therapy for nerve injury-induced pain in rats. PAIN 2019;160:210–23. 30188455 10.1097/j.pain.0000000000001395

[24] Thompson K Menzies S Muckenthaler M Torti FM Wood T Torti SV Hentze MW Beard J Connor J. Mouse brains deficient in H-ferritin have normal iron concentration but a protein profile of iron deficiency and increased evidence of oxidative stress. J Neurosci Res 2003;71:46–63. 12478613 10.1002/jnr.10463

[25] Toh WS Lai RC Zhang B Lim SK. MSC exosome works through a protein-based mechanism of action. Biochem Soc Trans 2018;46:843–53. 29986939 10.1042/BST20180079 PMC6103455

[26] Tsang A Von Korff M Lee S Alonso J Karam E Angermeyer MC Borges GL Bromet EJ de Girolamo G de Graaf R Gureje O Lepine JP Haro JM Levinson D Oakley Browne MA Posada-Villa J Seedat S Watanabe M Watanabe M. Common chronic pain conditions in developed and developing countries: gender and age differences and comorbidity with depression-anxiety disorders. J Pain 2008;9:883–91. 18602869 10.1016/j.jpain.2008.05.005

[27] Uttam S Wong C Amorim IS Jafarnejad SM Tansley SN Yang J Prager-Khoutorsky M Mogil JS Gkogkas CG Khoutorsky A. Translational profiling of dorsal root ganglia and spinal cord in a mouse model of neuropathic pain. Neurobiol Pain 2018;4:35–44. 30906902 10.1016/j.ynpai.2018.04.001 PMC6428075

[28] Wu X Cao Y Liu Y Zheng J. A new strategy for dietary nutrition to improve intestinal homeostasis in diarrheal irritable bowel syndrome: a perspective on intestinal flora and intestinal epithelial interaction. Nutrients 2024;16:3192. 39339792 10.3390/nu16183192 PMC11435304

[29] Xu R Pan Y Zheng K Chen M Yin C Hu Q Wang J Yu Q Li P Tai Y Fang J Liu B Fang J Tian G Liu B. IL-33/ST2 induces macrophage-dependent ROS production and TRPA1 activation that mediate pain-like responses by skin incision in mice. Theranostics 2024;14:5281–302. 39267790 10.7150/thno.97856 PMC11388077

[30] Zhang WJ Pi XW Hu DX Liu XP Wu MM. Advances and challenges in cell therapy for neuropathic pain based on mesenchymal stem cells. Front Cell Dev Biol 2025;13:1536566. 40061013 10.3389/fcell.2025.1536566 PMC11885280

[31] Zhang X Liu H Xiu X Cheng J Li T Wang P Men L Qiu J Jin Y Zhao J. Exosomal GDNF from bone marrow mesenchymal stem cells moderates neuropathic pain in a rat model of chronic constriction injury. Neuromolecular Med 2024;26:34. 39167282 10.1007/s12017-024-08800-6

[32] Zhao B Lin H Jiang X Li W Gao Y Li M Yu Y Chen N Gao J. Exosome-like nanoparticles derived from fruits, vegetables, and herbs: innovative strategies of therapeutic and drug delivery. Theranostics 2024;14:4598–621. 39239509 10.7150/thno.97096 PMC11373634

[33] Zhou XL Zhang CJ Peng YN Wang Y Xu HJ Liu CM. ROR2 modulates neuropathic pain via phosphorylation of NMDA receptor subunit GluN2B in rats. Br J Anaesth 2019;123:e239–48. 30916039 10.1016/j.bja.2018.08.025 PMC6676246

